# Intrathecal chemotherapy for the management of lymphoblastic lymphoma in a 4-year-old dog: a case report

**DOI:** 10.3389/fvets.2023.1209935

**Published:** 2023-09-05

**Authors:** Filipa Louise Simões Lyseight, Christophe Osterreicher Cunha Dupont, Giunio Bruto Cherubini

**Affiliations:** ^1^Oncology Service, Dick White Referrals, Part of Linnaeus Veterinary Limited, Cambridgeshire, United Kingdom; ^2^Neurology and Neurosurgery Service, Dick White Referrals, Part of Linnaeus Veterinary Limited, Cambridgeshire, United Kingdom; ^3^Department of Veterinary Sciences, University of Pisa, Pisa, Italy

**Keywords:** intrathecal chemotherapy, CNS lymphoblastic lymphoma, neuro-oncology, methotrexate, cytarabine arabinoside

## Abstract

Intrathecal chemotherapy is used in human medicine for the treatment or prophylaxis of CNS hematopoietic neoplasia. However, the clinical benefits in veterinary medicine have been scarcely documented. A 4-year-old male entire cross-breed dog presented with a 24-h history of severe lethargy, pelvic limb weakness, and urinary retention. Examination revealed generalized peripheral lymphadenomegaly, and the neurological findings were suggestive of a myelopathy in the region of T3-L3. Following the diagnosis of multicentric lymphoblastic B-cell lymphoma (stage Vb), a modified L-LOP with cytosine arabinoside was started, and complete clinical remission was achieved. After 4 weeks, there was acute neurological deterioration (spinal pain and proprioceptive deficits) without peripheral lymphadenomegaly. MRI findings and CSF analysis were consistent with meningeal and spinal cord lymphoma infiltration at the level of L3. Intrathecal chemotherapy (cytosine arabinoside and methotrexate) were administered in the cisterna magna with systemic dexamethasone and analgesia. Clinical signs were resolved within 24 h, and the patient remained asymptomatic for 3.5 weeks. After this period, CNS relapse (proprioceptive deficits and severe thoracolumbar pain) was suspected, and repeat intrathecal chemotherapy was declined. The patient was humanely euthanized 9 weeks after the initial diagnosis. This is the first report on the clinical benefit of intrathecal chemotherapy with a combination of methotrexate and cytarabine for the management of CNS lymphoma in dogs. Based on our case, intrathecal chemotherapy with methotrexate and cytarabine can induce a short-lasting CNS clinical remission (3 weeks).

## 1. Introduction

Primary or secondary central nervous system lymphoma (CNS) is clinically challenging to treat as cytotoxic medications that cross the blood–brain barrier (BBB) are limited (1–3). In humans, intrathecal chemotherapy has an important role in hematopoietic CNS neoplasia, particularly in treating children or older patients to minimize exposure to radiotherapy (4–8). Considering specifically lymphoblastic lymphoma, intrathecal chemotherapy composes most of the protocols prescribed in human medicine, mainly as a prophylactic measure due to the low incidence of CNS involvement during presentation (3–9%) (1, 3, 9, 10).

In dogs, Genoni et al. (11) documented a low complication rate (1/112) following intrathecal chemotherapy for the treatment of CNS neoplastic or inflammatory conditions. However, the literature on the clinical benefit of intrathecal chemotherapy for the management of CNS neoplasia in dogs is limited to six cases (12, 13). Of these, five had a multimodal approach (systemic chemotherapy, CNS radiotherapy, and intrathecal chemotherapy), and the sixth case was of a dog that had intrathecal chemotherapy and L-asparaginase. All these cases had intrathecal chemotherapy with a single agent, cytarabine arabinoside (11–13).

In this report, we have described the clinical benefit of cytarabine arabinoside and methotrexate administered intrathecally alongside systemic chemotherapy for the management of lymphoblastic lymphoma involving the CNS in a dog.

### 1.1. Case description

A 4-year-old male entire cross-breed dog presented with a 24-hour history of severe lethargy, pelvic limb weakness, and urinary retention. Previous clinical history was unknown as the patient had been recently rescued from aboard.

The dog was quiet, alert, and responsive on presentation. General clinical examination findings included generalized lymphadenopathy (right mandibular trilobed, 5 cm; left mandibular, 4.2 cm; right pre-scapular, 4.5 cm; left pre-scapular, 4.7 cm; and bilateral popliteal, 3 cm) and tachycardia (heart rate, 160 beats/min). On neurological examination, the patient was ambulatory paraparetic, had proprioceptive ataxia, absent paw replacement, and had markedly delayed hopping on the pelvic limbs with conserved segmental spinal reflexes. Pain was evident on cranial lumbar palpation. The cranial nerves and the remaining neurological examination were unremarkable. Neuroanatomical localization was compatible with T3-L3 myelopathy.

The patient deteriorated after admission to the clinic; he became depressed and developed spontaneous horizontal nystagmus with the fast left phase, right-sided vestibular ataxia, and reduced right menace response. Our main differential diagnosis was lymphoma with central nervous system involvement; however, the neurological signs could represent a different process, such as multifocal vascular, inflammatory, infectious, or less likely degenerative.

### 1.2. Diagnostic investigations, treatment, and outcomes

A complete blood count and blood film analysis (Table 1) showed pancytopenia characterized by mild regenerative anemia associated with moderate polychromasia and marked anisocytosis, moderate neutropenia with a low proportion of neutrophils with a toxic appearance, and moderate thrombocytopenia with a minimum manual estimate count of 110 x 10^9^/L. There were also circulating large, mononuclear, atypical cells (measuring x 1.5–2 diameter of a neutrophil), characterized by a large round nucleus containing finely stippled chromatin and variably prominent nucleoli (1–3 nucleoli) on a scant deep basophilic cytoplasm. The extended biochemistry panel and electrolytes did not show any significant or specific changes except for elevated C-reactive protein (77 mg/L) (ref.: <10). *Toxoplasma spp*. and *Neospora spp*. serologies were negative, and the urine analysis was unremarkable.

**Table 1:** Complete blood count results.

	**Day 0**	**Day 42**	**Day 44**	**Unit**	**Reference interval**
Haematocrit	0.28	0.28	0.24	L/L	0.37–0.55
Neutrophils	2.67	1.52	2.09	x 10^9^/L	3.0–11.5
Platelets	66	372	254	x 10^9^/L	200–500
Atypical cells	0.8	0.03	0.4	x 10^9^/L	0.0–0.01

Haematocrit, neutrophil, platelet, and atypical cell on admission (day 0), on the day of intrathecal chemotherapy (day 42) and 48 hours after intrathecal chemotherapy (day 44). On day 0 the anemia appeared regenerative (reticulocyte count 208.2 × 109/L, R.I.: 11–110) without evidence of haemolysis on blood film analysis. On day 42 and 44 there was no evidence of red blood cell regeneration and neutrophils did not exhibit toxic changes on blood film analysis.

Cytology analysis of peripheral lymph nodes identified a high number of monomorphic, large lymphoid cells (x2–3 small lymphocytes) with round nuclei with finely dispersed chromatin and prominent nucleoli. The cytoplasm was scant and basophilic. The mitotic count was high (8–10/5 x 60 HPF). The sample was also composed of small lymphocytes (<5%) and rare binucleated and mott cells. Flow cytometry of the peripheral lymph nodes was positive for CD79 (90%), CD45 (62%), and CD34 (96%) and negative for CD3, CD4, CD5, CD8, CD21, and MHCII.

The patient underwent thoracic radiographs (both DV and right lateral) to assess for intrathoracic lymphadenopathy. Additionally, a orthogonal radiographic lumbar spine study was performed to investigate the possible causes of urinary retention. Finally, an abdominal ultrasound was performed also under sedation. The diagnostic imaging findings included presence of mediastinal and intraabdominal lymphadenopathy, along with hepatomegaly characterized by heterogenous echogenicity. Splenomegaly was also observed, showing a diffuse mottled appearance. Furthermore, there was a small amount of pleural and peritoneal effusion.

Our findings were consistent with a multicentric lymphoblastic B-cell lymphoma (stage Vb) with suspected CNS involvement and bone marrow infiltration.

The patient was started on a modified L-LOP chemotherapy protocol (L-asparaginase, lomustine, vincristine, and prednisolone) with cytarabine arabinoside. The first treatments were administered, while the patient was hospitalized (Table 2, day 0). Although there was a moderate improvement of CNS signs with cytarabine (normal cranial nerves but persistent mild paraparesis with right-sided delayed postural reactions, considered a partial CNS response), he had persistent pancytopenia, which precluded further cytotoxic medication. Therefore, after being pre-medicated with chlorphenamine (0.4 mg/kg IV), L-asparaginase was administered (Table 2, day 0). During hospitalization, the patient also received analgesia for thoracolumbar pain (paracetamol 10 mg/kg PO q12h; gabapentin 10 mg/kg PO q12h; and buprenorphine 0.02 mg/kg IV q6h) and anti-nausea medication, as he showed signs of nausea (ondansetron 1 mg/kg PO q12h and maropitant 1 mg/kg IV q24h). An indwelling urinary catheter was placed to manage urinary retention, suspected to be secondary to thoracolumbar pain.

**Table 2:** Dose and schedule of systemic chemotherapy and glucocorticoids administered.

**Treatment schedule (days)**	**Chemotherapy agent/dose/route (Glucocorticoid/ dose/route/frequency)**	**Clinical systemic response**	**Clinical CNS response**
0	•Cytarabine arabinoside 300 mg/m^2^ IV infusion over 24 h •L-asparaginase 10,000 IU/m^2^ IM (Prednisolone 1 mg/kg PO q24h)	PD	PR
7	•Vincristine 0.7 mg/m^2^ IV (Prednisolone 1 mg/kg PO q24h)	CR	PR
14	•Lomustine 65 mg/m^2^ PO (Prednisolone 0.5 mg/kg PO q24h)	PD	CR
21	•(Prednisolone 0.5 mg/kg PO q24h)		
28	•Vincristine 0.7 mg/m^2^ IV (Prednisolone 1 mg/kg PO q24h)	PR^*^	CR
35	•Cyclophosphamide 200 mg/m^2^ PO (Prednisolone 1 mg/kg PO q24h)	PR^*^	PD
42	•Intrathecal chemotherapy: Methotrexate 2.5 mg and Cytarabine arabinoside 100 mg (Dexamethasone 0.2 mg/kg IV q24h, IV in clinic and PO after discharge)		CR
44	•Vincristine 0.5 mg/m^2^ IV (Dexamethasone 0.2 mg/kg PO q24h)	CR	
51	•Doxorubicin 1 mg/kg IV (Dexamethasone 0.1 mg/kg PO q24h)	CR	

Response was assessed 7 days after treatment except for Lomustine that was 14 days after. Clinical systemic response assessed according to RECIST criteria (21) by physical examination and circulating atypical cells. Clinical systemic response - complete remission (CR), palpable peripheral lymph nodes were > 1 cm length and hematology did not show circulating atypical cells; partial remission (PR), improvement of lymph node size >30% in comparison to baseline but > 1 cm length; PR^*^, palpable peripheral lymph nodes > 1 cm diameter but with persistent circulating atypical cells. Progressive disease (PD), increase in size of palpable peripheral lymph nodes and/or progressive atypical cells circulating. Clinical CNS response was determined by clinical history and neurological examination. Clinical CNS response was not considered to be due to systemic chemotherapy on weeks 6 and 7 as the patient had received ITC. CNS CR, absence of neurological deficits and thoracolumbar pain. CNS PR, persistent neurological signs but improved from previous; CNS PD, progressive neurological signs.

The patient was reassessed on a weekly basis for the continuation of the prescribed systemic chemotherapy protocol (Table 2). The clinical systemic response was assessed by measuring palpable peripheral lymph nodes and quantifying circulating atypical cells. The central nervous system response was measured by an improvement in neurological deficits and thoracolumbar pain. He had intermittent paraparesis during the first 2 weeks, but the remaining neurological examination was unremarkable. Analgesia for thoracolumbar pain was discontinued in Week 3. On day 28, there was progressive peripheral lymphadenopathy, and atypical cells were found circulating in the blood. As a rescue treatment, a CHOP-based protocol (cyclophosphamide, vincristine, doxorubicin, and prednisolone) was initiated. Despite variable responses to treatment (Table 2) during this period, the patient maintained a good quality of life without the side effects of chemotherapy.

**Figure 1:**
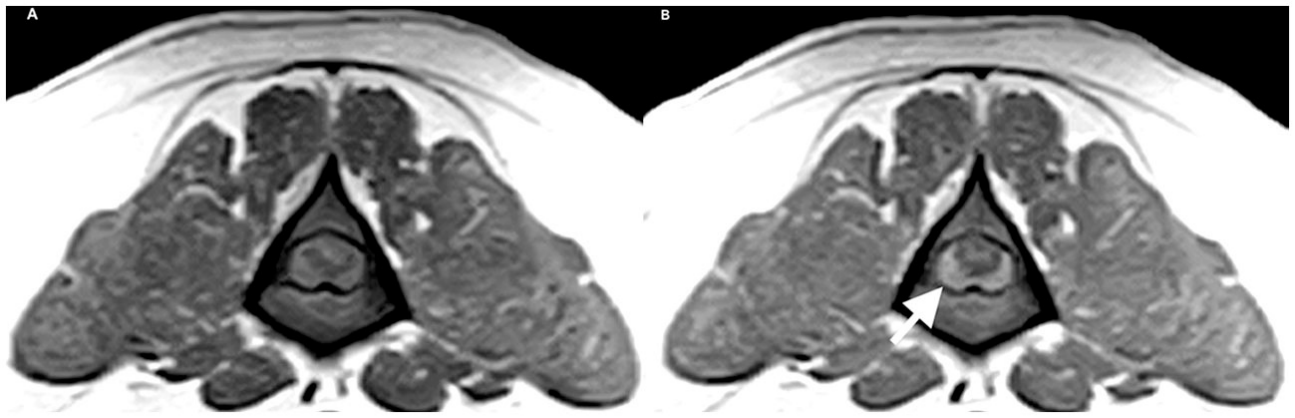
Transverse MRI of L3. **(A)** T1W and **(B)** T1W+C. White arrow showing a right 291 lateralized, contrast enhancing extramedullary compression.

The patient presented as an emergency 5 days after receiving cyclophosphamide. The dog showed severe thoracolumbar pain and bilateral hind limb proprioceptive deficits but without peripheral lymphadenopathy. To investigate the condition, various tests were conducted, including hematology and an extended biochemistry panel. Additionally, an MRI of the T3-S3 region was performed using sagittal T2W and STIR sequences, as well as transverse T2W, T1W, and T1W + C sequences). Furthermore, a CSF analysis was conducted, involving cytology and biochemistry, obtained from the cisterna magna. The MRI revealed compression of the spinal cord at the L3 level due to an interspersed, slightly right-sided, and ventral extradural material. The abnormal material observed in the spinal cord was slightly hyperintense on T2W and T1W MRI sequences to the spinal cord gray matter, and it exhibited clear contrast enhancement (Figure 1). This material caused compression at the L3 level, and, extending cranially, the spinal cord showed mild intramedullary patchy hyperintensity on both T2W and T1W images but without contrast enhancement. The CSF analysis revealed significant abnormalities, with a nucleated cell count of 1,900/uL (R.I: 0–5) and a protein of 0.34 g/L (R.I: 0.14–0.30). The cytology of the CSF also showed an abundance of atypical large lymphoid cells with irregularly round nuclei containing dispersed fine chromatin and several nucleoli, along with a small amount of basophilic cytoplasm. The presence of a high number of mitotic figures was also evident. These findings were consistent with meningeal and spinal cord lymphoma infiltration at the level of L3.

In view of this diagnosis, intrathecal chemotherapy was recommended. Under general anesthesia, the patient was placed in right lateral recumbency, and following the aseptic preparation of the area, 1 mL of CSF was retrieved from the cisterna magna. Through the same needle used for CSF collection, we administered 100 mg of cytarabine arabinoside (100 mg/mL solution) and 2.5 mg of methotrexate (2.5 mg/mL, 25 mg/mL diluted in 0.9% sodium chloride) intrathecally. The injection was administered over the course of 1 min. The patient tolerated the procedure and recovered from general anesthesia uneventfully. He received a single dose of intravenous dexamethasone and continued the same dose orally for 7 days (0.2 mg/kg q24h IV/PO), amantadine hydrochloride (5 mg/kg PO q12h), and gabapentin (10 mg/kg PO q12h). As the patient had afebrile neutropenia during intrathecal chemotherapy (Table 1, day 42), prophylactic cefalexin (10 mg/kg PO q8h) was started according to the practice guidelines.

The dog remained hospitalized for 48 h for monitoring, and no complications were noted. Neurological clinical signs resolved within 24 h of treatment administration. Three days after intrathecal chemotherapy, systemic chemotherapy was continued as persistent atypical cells were circulating (Tables 1, 2, day 44). This corresponded to an overall delay of 2 days from the scheduled protocol. The dexamethasone dose was reduced to 0.1 mg/kg q24h PO, and cefalexin was discontinued after 9 days when resolution of neutropenia was documented (neutrophil count 3.32 × 10^9^/L, R.I.: 3.0–11.5). During these assessments, the neurological examination remained unremarkable. 7 days after the administration of doxorubicin a reassessment confirmed a persistent complete clinical remission, and no neurological signs were reported or observed.

Twenty-five days after ITC, the patient presented with a relapse of neurological signs (severe thoracolumbar pain and hind limb deficits). There was no evidence of peripheral lymphadenopathy. A repeat ITC was declined. The dexamethasone dose was re-increased to 0.2 mg/kg PO q24h from 0.1 mg/kg PO q24h), and analgesia with amantadine hydrochloride (5 mg/kg PO q12h), paracetamol (10 mg/kg PO q12h), and gabapentin (10 mg/kg PO q8h) were introduced but did not have any clinical benefits. The patient was humanely euthanized after 48 h, 9 weeks after the initial presentation.

## 2. Discussion

Methotrexate and cytarabine arabinoside are examples of chemotherapy agents with proven clinical benefits in canine lymphoma and penetration of the BBB when administered intravenously (14–17). The diffusion of medications through the BBB is influenced by their molecular weight, ionization at physiological pH, protein binding, liposolubility, and interaction with MDR1 receptors (18). In human medicine, some will advocate for the use of high doses of methotrexate (e.g., 6–8 g/m^2^) systemically instead of intrathecal administration (6, 19, 20). However, such doses in dogs are associated with high toxicity, and this is not a treatment option (16). Cytarabine is thus administered intravenously as part of multi-agent protocols for the management of CNS neoplasia in dogs (17). In the case described, the response to cytarabine and prednisolone was mild and of short duration. Lomustine (cytotoxic agent with CNS penetration) was considered to induce CNS remission; however, our patient developed progressive peripheral disease. Therefore, at the time of the diagnosis of CNS relapse in the current case, intrathecal administration of chemotherapy was the best treatment option available. A standard dose of 100 mg of cytarabine and 2.5 mg of methotrexate is recommended. Dose adjustments, hypothetically extrapolating from human medicine, may be required in younger patients as the CSF's volume remains stable from the age of 3 years and is not influenced by body weight. Genoni et al. (11) administered the standard dose to patients aged 7 months to 14 years without significant complications in younger patients or those with a lower surface area (ranging from 3.5 to 53 kg). However, in cats, the dose of cytarabine was 50 mg and methotrexate 2.5 mg (11). Based on the information available, no dose adjustments are recommended based on age or body weight in dogs.

As previously described, the combination of cytarabine arabinoside and methotrexate administered intrathecally is well tolerated, and the clinical decision was made to use both medications (11). LaRue et al. (12) described a case of relapsed CNS lymphoma treated with ITC with cytarabine arabinoside only, and no response was reported (euthanized after 3 days). The other five cases reported received additional local treatment, including CNS radiotherapy, which led to a rapid improvement of clinical signs and clearance of CSF lymphoid neoplastic cells between 2 and 10 days later (12, 13). One case received six ITC treatments (2 x weekly) and six CNS RTs (Monday, Wednesday, and Friday) alongside systemic chemotherapy and lived for 84 days (13). A second case lived for 286 days after receiving ITC, radiotherapy (total dose of 44 Gy), and systemic chemotherapy (CHOP and rescue CCNU). However, information about the treatment schedule and response is not described. The remaining three cases were euthanized within 10 days due to neurological deterioration or suspected adverse effects of systemic chemotherapy (13). This combination of local therapy seems to induce rapid clinical remission, but radiotherapy was not available at our hospital. In our case, CNS clinical signs resolved within 24 h after ITC, and the response lasted for 3.5 weeks. As described in the human literature, repeat administrations may have been required for a sustained response.

Of the six dogs with CNS lymphoma treated with ITC reported in the literature, one was believed to have died due to tentorial herniation secondary to ITC (13). As suggested by the authors and described in the human literature, pre-treatment (for example, glucocorticoids) is essential for decreasing CSF pressure and theoretically decreasing this risk. Our case did not have any complications but had been pre-treated for 5 weeks. Case selection for a low complication rate associated with ITC may be detrimental to a favorable outcome. Factors such as disease burden, pre-treatment, the volume of CSF removed, the volume of medication administered, and overall health for general anesthesia should be considered before ITC.

LaRue et al. (12) identified a longer survival time in dogs with CNS lymphoma if treated with a multimodal approach, particularly chemotherapy. As intrathecal chemotherapy is not associated with myelotoxicity, concurrent treatment with systemic chemotherapy can be administered to manage the extra-CNS disease. In our case, there was no evidence of myelotoxicity after 2 days, and the prescribed CHOP-based rescue protocol could be continued. At the time of euthanasia, the patient did not have peripheral lymphadenopathy. The difference between the improvement of CNS deficits and peripheral disease (lymphadenopathy and circulating atypical cells) in our case suggests that there may be a benefit in combining local treatment (CNS radiotherapy, ITC) with systemic treatment (3, 5, 8).

## 3. Conclusion

Intrathecal chemotherapy may be considered in the selected cases of CNS lymphoma refractory to systemic chemotherapy with the penetration of the BBB. The combination of methotrexate and cytarabine arabinoside administered intrathecally induced a rapid, short-lasting (3.5 weeks) improvement in clinical signs and was safe overall.

## Data availability statement

The original contributions presented in the study are included in the article/supplementary material, further inquiries can be directed to the corresponding author.

## Ethics statement

Written informed consent was obtained from the participant/patient(s) for the publication of this case report.

## Author contributions

Acquisition of data, analysis and interpretation of data, revising the article for intellectual content, and final approval of the completed article: FL, CD, and GBC. Drafting the article: FL. All authors contributed to the article and approved the submitted version.
